# AScall – Automatic Allele-Specific qPCR Analysis

**DOI:** 10.3389/fbioe.2020.00353

**Published:** 2020-04-22

**Authors:** Konstantin Blagodatskikh, Dmitrii Romaniuk, Dmitry Malko

**Affiliations:** ^1^Molecular Oncology Laboratory, Pirogov Russian National Research Medical University, Moscow, Russia; ^2^Laboratory for Transplantation Immunology, National Research Center for Hematology, Moscow, Russia

**Keywords:** R, Shiny, allele-specific PCR, SNP, qPCR, AS-PCR, MiHA, minor histocompatibility antigens

## Abstract

Minor histocompatibility antigens (MiHA) are critical elements for the immune response after allogeneic hematopoietic stem cell transplantation. They may cause both beneficial and detrimental effects in forms of graft-versus-tumor and graft-versus-host accordingly. MiHAs originate from donor-recipient discrepancies in single nucleotide polymorphisms, insertions, and deletions. To determine the genetic mismatches between a donor and a recipient, we have implemented a real-time PCR method in conjunction with allele-specific primers (AS-qPCR). The new approach allows for multiplexing up to 480 reactions per 96 well plate and differs from common qPCR based genotyping methods. Earlier, we have confirmed and published the AS-qPCR method, but standard software for qPCR analysis does not suit AS-qPCR data. Here we fill this gap and describe AScall – the interactive web application for the proposed genotyping method. With a convenient interface mimicking a regular qPCR machine interface, our tool allows batch qPCR data import via universal RDML format, amplification curves preprocessing, quality control, sample genotype calling, detailed results visualization, and report generation. We show the use of AScall for SNP and indel genotyping for the MiHA study, but anyone can use the application for an accordingly designed AS-qPCR experiment of their own. Genotyping was done manually and with AScall for 96 genomic DNA samples. AScall processed 4,800 qPCRs in 1.5 min, with only two genotype mismatches compared to manual analysis. It took 3 h for an experienced researcher for manual analysis. Source code is freely available for download at https://github.com/kablag/AScall. The tool is freely available on the web at the AScall server http://shtest.evrogen.net/AScall.

## Introduction

Modern hematology heavily uses molecular methods for diagnosis and treatment selection. In this paper, we focus on genotyping for minor histocompatibility antigens (MiHA) (Griffioen et al., 2016). MiHAs are polymorphic peptides, after hematopoietic stem cell transplantation (HSCT) they can induce an immune response if the donor and the recipient mismatched for MiHA coding polymorphisms (de Bueger et al., 1992). In particular condition, these peptides could render recipient cells foreign for donor immune cells and cause detrimental consequences – systemic immune reaction toward the patient cells (Shlomchik, 2007). If a MiHA expression restricted to hematopoietic tissue, the MiHA still renders host hematopoietic cells foreign for the graft, but this would lead to the elimination of residual pathogenic cell clone, as it is also of hematopoietic origin (Falkenburg and Warren, 2011). Also, the tissue-specific MiHA can be a target for cell therapy (Bleakley and Riddell, 2011). The majority of MiHAs originate from non-synonymous single nucleotide polymorphisms (nsSNP), which change the amino acid sequence of proteins. Genotyping MiHAs allows us to predict HSCT consequences and study plausible targets for cell therapy.

Most MiHAs are encoded by nsSNP and could be genotyped by a vast arsenal of SNP-genotyping methods such as allele-specific PCR (AS-PCR), analysis of restriction fragments length polymorphism (RFLP), high resolution melting PCR (HRM-PCR), qPCR with fluorescent beacons or fluorescent hydrolysis probes, single nucleotide extension, DNA sequencing, and microarrays.

AS-PCR is a PCR with one universal primer and a pair of allele-specific primers (ASP), which 3′ end is complementary to each SNP allele (Ugozzoli and Wallace, 1991). To determine an SNP genotype, two independent PCR reactions needed: one per SNP allele (Spierings et al., 2006). RFLP analysis based on the digestion of the PCR product containing SNP of interest by type II restriction endonuclease that recognizes only one of the SNP variants (Pierce et al., 2001). Both methods require agarose gel electrophoresis to detect the PCR product or the restriction fragments and make the allele call. These two methods could be multiplexed because of product length difference, although it complicates the interpretation of the results. Contrary, real-time PCR methods, like HRM-PCR, qPCR with an allele-specific beacon, or a hydrolysis probe, could be performed in one step with little hands-on time. The second set of techniques uses fluorescent dyes to visualize product accumulation. The lack of a PCR post-processing step is a significant advantage as it reduces both contamination risk and hands-on time. HRM-PCR is based on a precise measuring of fluorescence drop while melting short amplicons after PCR with an intercalating dye. States of the SNP will slightly differ in melting temperature and melting speed. The limitations of the method are that it requires proprietary software, it is hard to multiplex, it needs control reactions each time, its sensitivity depends on the SNP type, and it is prone to inaccuracies (Słomka et al., 2017). qPCR with beacon or hydrolysis probes uses oligonucleotides conjugated with fluorescent dyes and quenchers, these beacons or probes are complementary to the SNP site. For one SNP genotyping separate probes with different dyes are needed for each allele – the typing performed in one tube. Probes and beacons with different fluorophores allow to multiplex reactions, but most commercially available qPCR-based SNP genotyping kits designed to genotype one SNP per test, so each SNP requires a separate test. This method is the most common and qPCR detection systems software able to perform allele call for the method. Amongst chemistry needed for a qPCR fluorescent probes are the most expensive, and there are two of them in each genotyping reaction. Also, probes binding depends on the SNP allele and DNA context.

Sanger sequencing is the most accurate method, but it lacks a multiplexing possibility and performed in several steps, which can take up to 1 day. SNP genotyping could be scaled up by a single nucleotide extension reaction or even next-generation sequencing (NGS). Both latter methods take much time to perform, up to several days, but allow to genotype dozens to thousands of SNPs simultaneously. Although it is excessive for genotyping of a small set of known MiHAs, it could be used for the discovery of new MiHAs (Kawase et al., 2008; Bergen et al., 2010). SNP genotyping techniques were reviewed in Kim and Misra (2007). MiHAs genotyping was reviewed in Spierings and Goulmy (2007).

As number of clinically relevant MiHAs is currently below a hundred, but more than a dozen, it is impractical to use either one SNP per test methods or NGS and microarrays. Besides, because MiHAs presentation restricted by an HLA allele, it makes sense to develop genotyping panels grouped by HLA restriction. Some MiHAs appear due to indels, so the method of choice should also be capable of genotyping indels.

We picked AS-PCR variation conducted with hydrolysis probes in real-time (designated from here on as AS-qPCR) (Stadhouders et al., 2010). Our goal was to make a robust, multiplex, and cheap method, so we stick to AS-qPCR as a method of choice. Although we have to use two tubes for an SNP to separate two allele-specific reactions, we can set as many parallel reactions in a well as a qPCR machine optical system allows: each target is “color-coded” using a *gene-specific* hydrolysis probe with a fluorescent dye. The probe is universal for both tubes. The positive outcome of AS-qPCR for the allele is judged by the simple fact of successful qPCR – an increase in the fluorescence signal.

In the earlier study, we developed our AS-qPCR for by far the largest HLA-A^∗^02:01 restricted group of 20 known MiHA (Romaniuk et al., 2019). The proposed approach reveals full power, performed in a multiplex – four SNPs can be genotyped using two wells. Moreover, the example genotyping set has control gene reaction incorporated into each well. It shows any pipetting inaccuracy and allows DNA load normalization and well comparison without technical repeats. One test is for gene deletion, and in this case, both tubes have the same gene-specific reaction for the indel. Although our proposed MiHA genotyping method is easy to analyze by the “visual approach,” almost five hundred events possible per plate render analysis time-consuming and error-prone. So, to accompany the genotyping, in this article, we report AScall – the program to foster batch data analysis for the AS-qPCR method. In the example, we used all five detection channels of the CFX96 Touch real-time PCR detection system (Bio-Rad, United States): four channels for target polymorphisms and one channel for a control gene. In the example, AS-qPCR kit for 20 HLA-A^∗^02 restricted MiHAs, four hundred reactions run in parallel in a 96 well plate for eight DNA samples: for one control gene, 19 SNPs, and one gene deletion. The performance of our program was shown using 96 genomic DNA samples genotyped with the AS-qPCR method for this set of MiHAs and was compared to manual genotyping. AScall software allowed to reduce analysis time and showed comparable with manual genotyping results.

Detection systems for qPCR could be found in many scientific and diagnostic molecular biology labs, as qPCR is a fast and handy method for various applications. So, as an obvious consequence, there are many software tools for qPCR analysis, particularly for SNP genotyping. To our knowledge, all SNP calling tools for qPCR analyze data obtained by “one-tube” approach, an SNP is genotyped in one tube using two *allele-specific* hydrolysis probes with different fluorescent dyes, one SNP per well. Such an approach implemented in the CFX Manager software by the Allelic Discrimination plot. Also, these programs do not allow more sophisticated analysis with quality control for the experiment. The R language is widely used for processing qPCR data (Rödiger et al., 2015a; Burdukiewicz et al., 2018). However, ready-made solutions, such as Chainy (Mallona et al., 2017), do not correspond to our specific task. AScall program is not limited to the example genotyping panel but is readily applicable to accordingly designed primer sets and any number of reactions per well.

## Method (Including Any Code Description)

**FIGURE 1:**
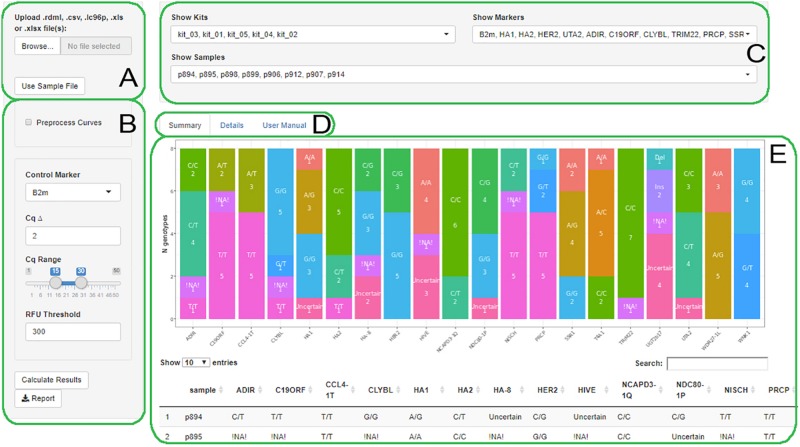
The AScall web interface. **(A)** File selector; **(B)** Settings; **(C)** Filters; **(D)** Summary, Details or User manual tabs; **(E)** The Summary tab view.

AScall is written in the R language with a graphical user interface based on the R Shiny library. Process of analysis consists of several steps: (1) data import from a qPCR machine via RDML format (Lefever et al., 2009); (2) optional qPCR fluorescent data preprocessing, otherwise the data will be used as a qPCR system software has processed it; (3) overall experiment quality control and individual sample control; (4) sample genotype calling; and (5) optional report generation using xlsx format. The graphical user interface consists of several visual blocks: (a) data import; (b) analysis settings; (c) filters; (d) summary, details, and help tabs selector; and (e) main window (Figure 1). The user manual is available from the help tab.

### Plate Setup

**FIGURE 2:**
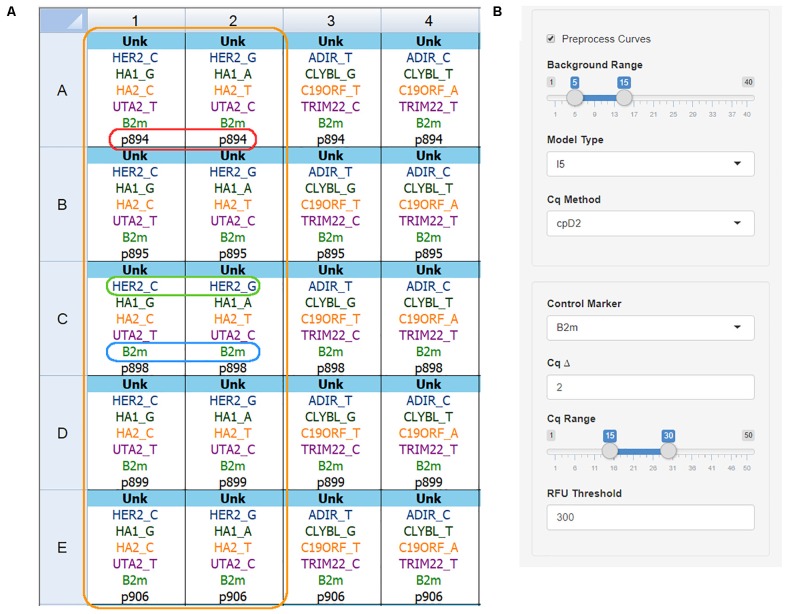
qPCR plate setup and analysis settings. **(A)** Plate setup view for the Bio-Rad CFX Manager v3.1 software showing correct name convention: orange box – one primer kit set for five DNA samples; red box – sample name is the same for both wells; green box – target name and allele name are separated by the underscore sign; blue box – control gene name is the same for all wells; **(B)** The AScall import and preprocessing options, no amplification range, curve fitting model, Cq determination method can be set for preprocessing.

Naming convention and file export are shown using Bio-Rad CFX Manager 3.1 as an example. Figure 2A depicts the correct plate setup for two primer kits. There are several naming rules for the data to be processed correctly:

• All tubes with one DNA sample must be **named equally**. Sample name **p894** (Figure 2A, red box): despite the different target names – sample names are equal. Same rule for replicates – any indices are not allowed.
• Target names for studied genes must contain **gene name** and **allele name** after **underscore** – GENENAME_ALLELENAME pattern. The green box in Figure 2A frames **HER2_C,** and **HER2_G** target names, where **HER2** is the gene name, and **C** or **G** are alleles. For indel targets use the plus sign (e.g., **UGT2b17_+)**. Use of indel target naming changes analysis: no amplification in a well is a deletion; amplification – insertion.
• The control gene name has to be the same for all wells, e.g., **B2m** (Figure 2A, blue box).
• No template controls must have an **NTC** sample type.
• The target name without an allele name is called a **marker**.
• AScall interprets all tubes with the same marker set as one **kit** (Figure 2A, orange box).

### Export Data From Bio-Rad CFX Manager

After the data file with a correctly set plate is ready, one can export data by **Export > Export RDML File > RDML v1.1** menu of the Bio-Rad CFX Manager software. Use appropriate steps for other qPCR detection systems software.

### Data Import

Data import was implemented with the RDML package (Rödiger et al., 2017). One or more files can be imported in a format supported by the RDML package: rdml, lc96p, xlsx, and some other. Only with correct sample naming AScall will work as an automatic genotype caller. If several files were loaded, all subsequent analyses carried out independently for each plate, with joined genotypes plot and data tables provided (control gene and preprocessing settings will be the same for all plates). The “Use Sample File” button runs the program test with the example file.

### PCR Curves Processing

PCR curves processing contains three steps: background signal subtraction via *CPP* function from *chipPCR* package (Rödiger et al., 2015b), PCR curve model fitting, and Cq calculation by *pcrfit* and *efficiency* functions from *qpcR* package respectively (Ritz and Spiess, 2008). Several settings available for processing (Figure 1B):

• **Control Marker** – select any detected marker as a *control marker* – the reaction that has to be positive in all wells;
• **Cq Δ** – maximum difference between Cq values for the corresponding wells for a heterozygous call;
• **Cq Range** – Cq range values for a reaction to be treated as positive;
• **RFU Threshold** – minimum end-point fluorescence signal for a reaction to be treated as positive.

Additional preprocessing options will be available if the “Preprocess Curves” checkbox ticked (Figure 2B):

• **Background Range** – cycles to calculate background signal (linear part of the curves before exponential growth) for all curves;
• **Model Type** – fits one of the models to the PCR data using (weighted) non-linear least-squares using *pcrfit* from qpcR package (Ritz and Spiess, 2008);
• **Cq Method** – calculates the quantification cycle with one of the methods: maximum of the first or second derivative curve, maximum of the efficiency curve, exponential region, 20% value of the fluorescence at second derivative curve maximum using *efficiency* function from qpcR package.

Preprocessing can be carried out for raw or proprietary software exported data. Preprocessing is an optional step, and it is only needed for raw data or if **Cq** values should be recalculated with the independent method, like a second derivative maximum.

**FIGURE 3:**
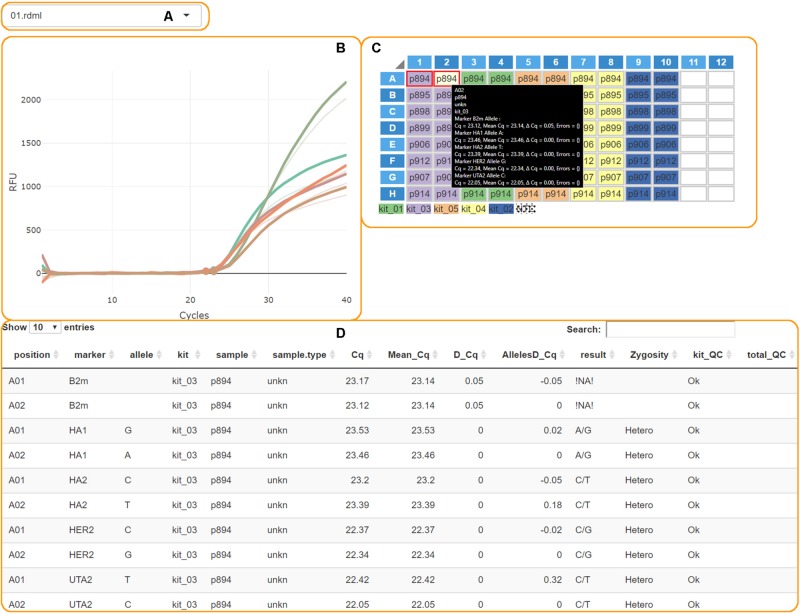
The AScall details tab. **(A)** File selector; **(B)** PCR curves; **(C)** Plate overview and well selector; **(D)** Details table.

The “Calculate Results” button finalizes the analysis. The genotypes plot with all allele counts and the summary table will appear. Processed PCR curves can be viewed in the details tab (Figure 3).

### Overall Experiment Quality Control and Individual Curve Control (QC)

These steps are conducted to check for possible experiment problems. The QC checkpoints are minimal fluorescence signal and Cq thresholds, the signal from NTC samples, a control gene signal, a Cq delta for paired wells. The QC results are provided in the table in the details tab (Figure 3D). The QC codes can be found in Table 1. AScall software adjusts the Cq delta slightly for target markers using corresponding Cq difference for the control gene for the pair of wells. If one or both AS-qPCR reactions for a marker are inside the Cq range, but the end-point fluorescence level is below the RFU threshold, then the call for the pair of wells would be “Uncertain.” The same call would be in the opposite case: Cq is out of the range, but the curve is above the RFU threshold.

**TABLE 1:** Quality check codes description.

QC name	Message
2*RFU_QC	“OK”
	“Low” – curve endpoint fluorescence signal is lower than “RFU Threshold”
2*ampStatus_QC	“OK”
	“NoAmp” – amplification for this curve is not detected
2*replicateMatch_QC	“OK”
	“Fail” – Cq difference between replicates bigger than “Cq Δ” option
2*noAmpNTC_QC	“OK”
	“Fail” – any NTC sample has positive amplification
2*ctrlMarker_QC	“OK”
	“Fail” – control marker does not have amplification in a well
2*allelesDeltaCq_QC	“OK”
	“Fail” – Cq difference between alleles of a marker is bigger than “Cq Δ” option

### Genotype Calling

**FIGURE 4:**
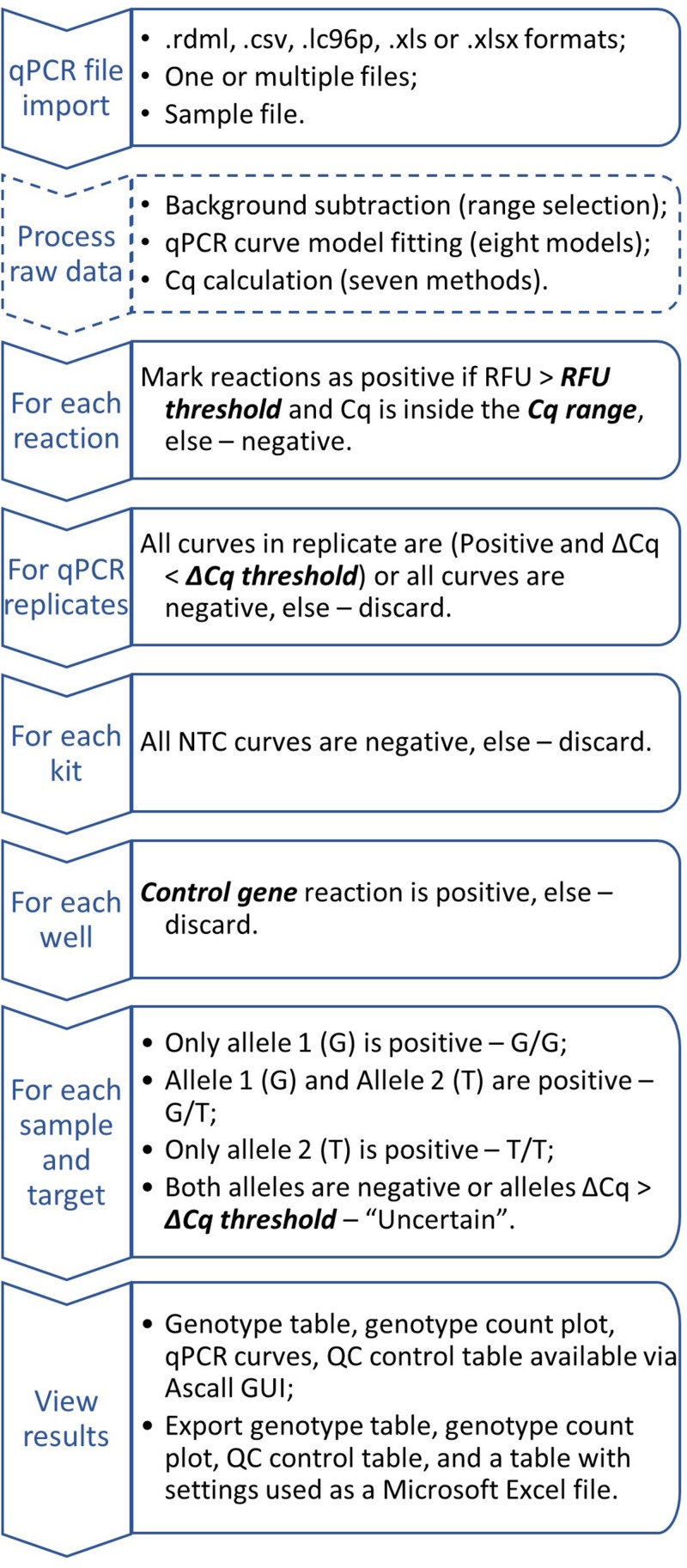
Generalized AScall analysis workflow. Optional steps dashed, user-controlled parameters are in bold italic.

It can be done only for samples passed QC: kit_QC == "Ok" & replicateMatch_QC == "Ok" & ctrlMarker_QC == "Ok". Then the result is a combination of alleles with ampStatus_QC == "Ok." For indel markers result will be insertion or deletion if ampStatus_QC == "Ok" or ampStatus_QC != "Ok", respectively. General workflow chart is shown in Figure 4.

### Visualization

There are three main elements of the program GUI (Figure 1).

### Global Filters

Global filters allow selecting individual samples, markers, or kits for viewing at details and summary tabs (Figure 1C).

### Summary View

This view shows all genotyping results for all loaded files as a bar plot and a table (Figure 1E). The table represents genotyping results by sample. Bar plot allows to overview results by markers: the x-axis is a marker, and the y-axis is genotype count.

### Details View

The details tab provides access to additional per plate details about PCR curves and analysis (Figure 3). Plots and plate layout were done using shinyMolBio library (Blagodatskikh, 2019). It consists of:

• **File selector** – one can switch uploaded data files by this element (Figure 3A).
• **PCR curves** – created by shinyMolBio:renderAmpCurves() function (Figure 3B). Curves are colored by *marker*.
• **PCR plate** – created by shinyMolBio:pcrPlateInput() function (Figure 3C). Wells are color-coded by *kit*; dotted wells contain NTC (see the legend under the plate). Selected wells have a red border, and *on-hover* well has a light-yellow background. If any curves were selected – only corresponding curves would be on the PCR curves plot, and the *on-hover* curves are solid while the others are transparent. *On-hover* well provides additional info inside a box.
• **Details table** – shows information about every PCR curve, including genotyping results and QC (Figure 3D).

### Report

After analysis, the summary report could be generated by clicking the “Report” button. The report function allows exporting genotyped data after single or batch file analysis. The report consists of:

• Genotyping results table
• Genotypes count plot
• QC table
• Used settings list

Report generation is carried out by *openxlsx* package in the xlsx format compatible with Microsoft Excel software (Walker, 2019).

### Performance Assessment

The genotyping was done as described earlier (Romaniuk et al., 2019). Briefly, primers and probes for genotyping of 20 polymorphisms split into five kits. Each kit consists of two mixes of primers either for reference or alternative alleles of the SNPs or indel as marked by Human genome assembly GRCh38^1^. Each mix also has the control gene primers. Primer mixes and 5× qPCR-HS ready qPCR mix (EvroGen, Russia) were used to prepare stock genotyping solutions. Stocks were validated using control plasmid mixes. Peripheral blood samples for genotyping obtained from volunteers. DNA extracted using the Wizard Genomic DNA Purification Kit (Promega, United States). Altogether, 96 genomic DNA samples, separated into twelve plates, were genotyped. Samples with low DNA concentration were intentionally left to check AScall performance. qPCRs performed using the CFX96 Touch system (Bio-Rad, United States). The proprietary CFX Manager 3.1 software used for manual analysis and data export in the RDML data format version 1.1, one file per plate. For manual analysis, the baseline interval was 5–15 cycles, cycle thresholds set automatically. Heterozygous call for a gene was made if both tubes were positive for the control gene and the allele-specific reactions. The homozygous call was made, if only one allele-specific reaction were positive, while controls are still positive for both tubes. Automated analysis using AScall was performed for twelve rdml files simultaneously, with the Cq delta set to 3.5, Cq range set to 15–25 cycles, and RFU threshold of 200. Hands-on time measured for manual and automated data processing performed by an experienced user, for one or 12 data files (plates), respectively. Manual genotyping was done blindly and independently. The generated report was used to compare manual and automated analysis.

## Results (Including Examples of Use and Limitations)

### The AScall Software

AScall is a user-friendly, open-source tool for the allele-specific qPCR analysis written with the R language and R Shiny library. It can accept data from a wide range of qPCR machines, either raw or preprocessed. File formats supported by the AScall include, but not limited to, rdml, lc96p, xlsx. A batch analysis is also supported. AScall is a cross-platform web-based tool. It can be used online at http://shtest.evrogen.net/AScall/or implemented locally. The source code of AScall is written according to the Model-View-Controller programming pattern so that it can use a separate server for faster data computation. AScall has a convenient graphical user interface and straightforward operational pattern. We tried to make AScall resemble a qPCR detection system’s software so that any user can implement the same analysis logic as usual. AScall is not bound to the specific kit and can be used with similar homemade kits and with any number of color channels (primer sets), and samples.

The AScall user interface separated into three visual blocks: data import and processing, visualization of the results, and filters to select desired markers, kits, or samples (Figure 1). The AScall software can import several files for batch analysis and allows setting desired data processing parameters to finetune genotype call. It also allows recalculating Cq using eight curve-fitting models, seven Cq computation methods, and a readjusted background range.

For convenience, processed result visualization separated into two tabs: summary (Figure 1E) and details (Figure 3). The first one shows overall statistics as a genotype barplot and genotype table for all processed files. The details tab is for by-well details, fluorescent curves, genotypes, and possible quality control errors. Plate overview provides all necessary actions for curve selection and highlighting as a standard qPCR software do. The individual curves view is convenient for a quick check for problems that may appear during qPCR (Figure 3). The filters used together with file and well selectors at the details tab can help to narrow study results. Also, there is a separate tab with the user manual for convenience. Another option to view results is report generation. It becomes available after computation and generates Microsoft Excel-compatible file with genotyping results, genotype count plot, QC control table, and a table with settings used. For batch import, a joint summary and a joint report will be provided. An overview of the AScall software workflow is shown in Figure 4.

### AS-qPCR Data Analysis With AScall

For AScall software to work as designed, one should consider a strict naming convention. The target name must have two parts: an SNP or indel name, and the allele name or plus sign for the indel, separated by the underscore symbol (Figure 2). Sample replicates, positive and none template controls are optional but must follow naming conventions. The easiest way to use the AScall tool is by exporting AS-qPCR data files from a qPCR detection system software as RDML v1.1 files and importing them into the AScall tool. Overall experiment quality control and individual curve control (QC) conducted to check for possible experiment problems. The QC checkpoints are minimal fluorescence signal and Cq thresholds, the signal from NTC samples, the control gene signal, and Cq delta. The QC results provided in the table of the details tab (Figure 3D). Genotype calling can be done only for samples passed QC. For an SNP, the genotype is determined by the rule of thumb: if only one allele-specific reaction is positive – the sample is homozygous for this marker; positive results for both allele-specific reactions – the sample is heterozygous. For an indel, both wells should be positive or negative. The MiHA genotyping obtained using AScall was implemented to select donors for *in vitro* T cell studies.

### AScall Comparison With Manual Genotyping

Side by side comparison of manual and automated genotyping was made for 96 DNA samples tested for 20 markers. AS-qPCRs were set without DNA concentration adjustment. Altogether, 4,800 qPCRs have been processed both manually and with AScall, with the total possible number of genotype calls of 1,920 for target genes. Manual genotyping was done blindly and independently and took approximately 15 min per plate, 3 h altogether, while automated analysis with ready rdml files took only one and a half minutes, with most of the time spent for file upload. During manual analysis, two samples (40 genotypes) and 15 scattered genotypes were excluded due to low control gene signal or low target gene signal, 1,865 genotypes left in total. AScall software excluded 88 reactions as not available and 73 as “Uncertain” due to more stringent QC. First of all, each pair of wells is checked for control gene reactions: both should be positive and end-point fluorescence should be above the set threshold, Cq difference for control reactions between respective wells should be less than the preset value, and the Cq should be inside the Cq range. Also, the control gene end-point signal shows plausible evaporation – well-outlier is excluded altogether with the paired well. Some of these reactions can still be assessed manually with caution. Also, to exclude false positives, the same check algorithm was applied to target genes. AScall made 1,759 genotype calls. There were two mismatches with manual typing. One automated genotype call made for the weak signal well pair, the genotype for these wells was not determined during manual assessment. The other genotype mismatch was also due to low signal, and we cannot say it was wrong, as a low signal can also lead to a wrong reaction interpretation by a researcher. During extensive testing with different settings, only some wells with a weak signal generated false results, so for fine-tuned AS-qPCR testing, AScall software can be a robust and reliable tool, and default setting will be sufficient.

## Discussion (Including Scalability and Limitations)

As far as we know, there are no other software tools for automated analysis of multiplexed allele-specific qPCR wherein alleles separated into different wells and color channels and with introduced control for DNA presence in a well. Software supplied with qPCR detection systems offers only simple genotyping with two detection channels used per well, gene expression analysis, or standard calculation of a threshold cycle. Thus, a user of the AS-qPCR method may get threshold cycles from the proprietary software but must make labor and error-prone genotyping manually. AScall software overcome this issue, as it specifically designed for the AS-qPCR method. AScall has a smooth, user-friendly interface, anyone, who knows how to work with a qPCR software can handle AScall.

DNA samples of mediocre quality were the main reason for the target genotype to be marked as “Uncertain” by the software. The manual genotyping could overcome this issue only to a certain extent, as human interpretation can lead to erroneous results.

The tool is written in language R with Shiny for a web interface so it can be easily changed for user-specific needs. It can run at a server or a local computer. Although we tried to predict as many qPCR errors as possible, the tool may still be prone to genotyping errors caused by weak fluorescent signals or wrong Cq interpretation by proprietary software. This type of mistake can be easily excluded by eye analysis. Also, AScall can recalculate Cq with many different methods if needed. Maybe the implementation of neuronal networks can be beneficial for AScall software.

Overall, AScall is documented, freely available, and open source. Thus, our software can be part of an analysis pipeline corresponding to the Minimum Information for Publication of Quantitative Real-Time PCR Experiments (MIQE) guidelines (Bustin et al., 2009).

## Data Availability Statement

The data files used for the study are at the GitHub https://github.com/kablag/AScall.

## Author Contributions

DR and KB designed the software. KB made the GUI and web server. DR, DM, and KB tested the software and wrote the manuscript.

## Conflict of Interest

The authors declare that the research was conducted in the absence of any commercial or financial relationships that could be construed as a potential conflict of interest.

## Footnotes

1 http://ensembl.org

## Software Information

Project link:

https://github.com/PCRuniversum/shinyMolBio

Implementation:

http://shtest.evrogen.net/AScall

Operating system:

Platform independent

Programming language:

R

License type:

The AScall software is distributed under MIT license.
